# Fully‐Constrained Variable Projection for Water‐Fat Models

**DOI:** 10.1002/mrm.70428

**Published:** 2026-05-10

**Authors:** Carl Ganter, Jonathan Stelter, Louis Peyratoux, Dimitrios C. Karampinos, Oliver Bieri

**Affiliations:** ^1^ Department of Diagnostic and Interventional Radiology, TUM University Hospital Technical University of Munich, TUM School of Medicine and Health Munich Germany; ^2^ Laboratory of Magnetic Resonance Imaging Systems and Methods École Polytechnique Fédérale de Lausanne (EPFL) Lausanne Switzerland; ^3^ CIBM Center for Biomedical Imaging Lausanne Switzerland; ^4^ Division of Radiological Physics, Department of Radiology University Hospital Basel Basel Switzerland; ^5^ Department of Biomedical Engineering University of Basel Allschwil Switzerland

**Keywords:** constrained phase, Dixon, multi‐echo GRE, variable projection, water‐fat

## Abstract

**Purpose:**

Variable projection (VARPRO) is an effective technique to reduce the dimensionality of least squares optimization problems through the elimination of linear parameters. For water‐fat models, constrained VARPRO variants have been proposed in the literature to ensure that water and fat components are aligned at TE = 0, thereby preventing overfitting of data. These models, however, still allow for anti‐parallel orientation of water and fat and therefore remain potentially vulnerable. An alternative, fully‐constrained VARPRO formulation is derived, which resolves these limitations without compromising dimensionality reduction and/or numerical performance.

**Theory and Methods:**

The linear coefficients are redefined according to the physical constraints, thereby introducing the fat fraction f as an additional nonlinear parameter. To remove the latter and regain the original VARPRO dimensionality, it is shown that the maximum likelihood (ML) estimate of f can be expressed as a closed‐form function of local off‐resonance frequency ω and relaxation rate $R_{2}^{*}$.

**Results:**

For challenging data, estimation of ω, $R_{2}^{*}$ and f becomes more stable using the proposed fully‐constrained VARPRO method.

**Conclusion:**

Fully‐constrained VARPRO enables more reliable local parameter estimation, while preserving the efficiency of existing approaches.

## 1 Introduction

The ability to reliably separate water and fat components is important for many applications, such as water/fat imaging [1], quantification of the proton density fat fraction (PDFF) [2], $R_{2}^{*}$‐mapping [3], $B_{0}$‐mapping [4] or quantitative susceptibility mapping (QSM) [5]. To this end, Dixon techniques mostly analyze the FID of multi‐echo gradient echo (GRE) sequences, exploiting the fact that water and fat differ in their spectral composition.

Data fitting usually involves the minimization of a cost function

(1) $$ℒ:=\underset{\rho }{\sum }\chi_{\rho }^{2}+ℛ,$$

which combines data consistency $\chi_{\rho }^{2}$ at location ρ and regularization ℛ. The latter, if present, most commonly [6, 7, 8, 9] promotes smoothness of the fitted field map.

To set up $\chi_{\rho }^{2}$, the typical approach is to choose a fixed m‐peak spectral fat model and to assume a common $R_{2\rho }^{*}$ for all water and fat peaks.

This leads to local cost functions of the form

(2) $$\chi_{\rho }^{2}\left(\omega_{\rho },\;R_{2\rho }^{*},\;c_{w\rho },\;c_{f\rho }\right),$$

where $\omega_{\rho }$ denotes the local resonance frequency and the complex linear coefficients $c_{w\rho }$, $c_{f\rho }$ respectively represent the initial weights and phases of water and fat at TE=0.

While early data modeling [10, 11] did not impose any restrictions on the linear coefficients $c_{w\rho }$ and $c_{f\rho }$, constraining the initial phase to a common value

(3) $$\mathrm{Arg}\left[c_{w\rho }\right]\overset{!}{=}\mathrm{Arg}\left[c_{f\rho }\right]=:\phi_{\rho },$$

was later found important [12, 13] to reduce problems due to overfitting, such as PDFF bias, observed at low SNR [14].

The complexity of the optimization can be reduced by variable projection (VARPRO) [15], replacing (2) by modified cost functions

(4) $$\chi_{\rho }^{2}\left(\omega_{\rho },\;R_{2\rho }^{*}\right).$$

Thereby, the linear coefficients $c_{w\rho }$, $c_{f\rho }$ no longer appear as variable parameters, but can simply be calculated after the optimization.

However, complying with the aforementioned phase constraint now becomes a challenge, since VARPRO cannot enforce the in‐phase condition (3). Bydder et al. [16] therefore added $\phi_{\rho }$ as an additional nonlinear parameter in a modified VARPRO cost function

(5) $$\chi_{\rho }^{2}\left(\omega_{\rho },\;R_{2\rho }^{*},\;\phi_{\rho }\right),$$

such that the linear weights of water and fat become real. They were further able to derive a closed‐form solution

(6) $$\phi_{\rho }=\phi_{\rho }\left(\omega_{\rho },\;R_{2\rho }^{*}\right),$$

which can be calculated efficiently and, after inserting into (5), thereby regained the original form (4) of the VARPRO cost function. (Strictly speaking, the derivation in [16] was limited to the special case $R_{2\rho }^{*}=0$, since only two echo times were considered. The derived expressions in [16] equally apply to the more general case, though.)

As will be shown below, the described inclusion of $\phi_{\rho }$ as a nonlinear parameter to VARPRO does not guarantee that (3) holds, though. In fact, while restricting the general phase orientation, this augmented VARPRO variant still allows for anti‐parallel orientation of water and fat at TE=0

(7) $$\mathrm{Arg}\left[c_{w\rho }\right]=\mathrm{Arg}\left[c_{f\rho }\right]\pm \pi ,$$

since the linear real coefficients may have opposite signs. Allowing for these unphysical solutions may therefore still cause overfitting.

In this article, we will derive an alternative, fully constrained VARPRO variant, which is guaranteed to satisfy the in‐phase condition (3). To this end, we will work with the fat fraction $f_{\rho }$ instead of $\phi_{\rho }$ as an additional nonlinear parameter, leading to a VARPRO cost function

(8) $$\chi_{\rho }^{2}\left(\omega_{\rho },R_{2\rho }^{*},f_{\rho }\right).$$

Like in [16], we will then derive a numerically efficient analytical solution

(9) $$f_{\rho }=f_{\rho }\left(\omega_{\rho },\;R_{2\rho }^{*}\right),$$

to regain the desired form (4).

This is followed by an investigation, under which conditions this proposed improvement becomes relevant.

## 2 Theory

### 2.1 Free Weights: FW‐VARPRO

In the following, we will focus on a fixed location and drop any explicit dependence on ρ.

Let us consider a set of complex signals $s_{j}$ from a multi‐echo RF spoiled gradient echo (GRE) sequence, acquired at echo times $t_{j}$ (not necessarily equidistant). For water‐fat mixtures, we assume $s_{j}={ŝ}_{j}+\eta_{j}$, where $\eta_{j}$ denotes complex (white) noise and the signal model is given by

(10) $${ŝ}_{j}:=\left(c_{w}+c_{f}\;\beta_{j}\right)\;e_{j},$$

(11) $$e_{j}:=e^{i\;\omega \;t_{j}}\;e^{-R_{2}^{*}\;t_{j}}.$$

The m‐peak fat model $\beta_{j}$ is specified as

(12) $$\beta_{j}:=\overset{m}{\underset{k=1}{\sum }}\;\alpha_{k}\;e^{i\;\omega_{k}t_{j}}\;\overset{m}{\underset{k=1}{\sum }}\;\alpha_{k}=1,$$

with chemical shifts $\omega_{k}$ and relative amplitudes $\alpha_{k}>0$ assumed to be known.

In (11), ω denotes the local off‐resonance (angular) frequency (corresponding to water), and we adopted the common assumption that all peaks (including water) decay with the same rate $R_{2}^{*}$. No phase constraint is assumed for the independent complex linear coefficients $c_{w},\;c_{f}\in \mathbb{C}$.

With the cost function $\chi^{2}$ defined by

(13) $$\chi^{2}\left(\omega ,\;R_{2}^{*},\;c\;\right):=\underset{j}{\sum }\;{\left|\;s_{j}-{ŝ}_{j}\;\right|}^{2},$$

($c:={\left[c_{w}\;c_{f}\right]}^{T}$), the local maximum likelihood (ML) estimators are obtained by

(14) $$\hat{\omega },\;{\hat{R}}_{2}^{*},\;\hat{c}=\text{argmin}\;\chi^{2}\left(\omega ,\;R_{2}^{*},\;c\right).$$

The dimensionality of the minimization problem (14) can be reduced with variable projection (VARPRO) [15]:

We write (13) in vector notation

(15) $$\chi^{2}=:{\left\|\;s-A\;c\;\right\|}_{2}^{2},$$

with the two‐column matrix

(16) $$A_{j1}:=e_{j}\;A_{j2}:=\beta_{j}\;e_{j},$$

and note that $\hat{c}$, as defined in (14), can be explicitly calculated for any *fixed* ω and $R_{2}^{*}$

(17) $$\hat{c}\;\left(\omega ,\;R_{2}^{*}\right)={\left(A^{*}A\right)}^{-1}A^{*}\;s=:B^{-1}\;b.$$

($A^{*}$ denotes the conjugate transpose of A. We further introduced the shorthands $B:=A^{*}A$ and $b:=A^{*}s$.)

Inserted on the RHS of (14), we then obtain the VARPRO cost function

(18) $$\begin{array}{ll} \chi^{2}\left(\omega ,R_{2}^{*}\right) & :=\chi^{2}\left(\omega ,\;R_{2}^{*},\;\hat{c}\left(\omega ,\;R_{2}^{*}\right)\right)\\ & =s^{2}-b^{*}B^{-1}\;b, \end{array}$$

for which the minimization (14) simplifies to

(19) $$\hat{\omega },\;{\hat{R}}_{2}^{*}=\text{argmin}\;\chi^{2}\left(\omega ,\;R_{2}^{*}\;\right).$$

With these estimators, we then directly obtain those of the linear coefficients

(20) $$\hat{c}=\hat{c}\;\left(\hat{\omega },\;{\hat{R}}_{2}^{*}\right).$$

But there is a problem with this procedure: With all $\alpha_{k}$ real and positive, every fat peak must obviously have the same phase (defined by $c_{f}$) at t=0, regardless of its chemical shift $\omega_{k}$. Consistency, therefore, demands that the same applies to the water peak as well, which means that $c_{w}$ and $c_{f}$ must have the same phase.

While a minimization of (14) subject to this constraint is possible, we unfortunately cannot use VARPRO for this, since the linear coefficients are simply determined by (20) and there is no way to enforce that $c_{w}$ and $c_{f}$ have equal phase.

### 2.2 Real Weights: RW‐VARPRO

To this end, Bydder et al. [16] and Stinson et al. [8] aligned water and fat components via

(21) $$c_{w}=:e^{i\;\phi }\;r_{w}\;c_{f}=:e^{i\;\phi }\;r_{f},$$

with real‐valued water and fat components $r_{w},r_{f}\in \mathbb{R}$, such that (15) becomes

(22) $$\chi^{2}={\left\|\;s-e^{i\;\phi }\;A\;r\;\right\|}_{2}^{2}.$$

VARPRO with respect to r then leads to the solution [16]

(23) $$\hat{r}\;\left(\omega ,\;R_{2}^{*},\;\phi \right)=\text{Re}{\left[B\right]}^{-1}\;\text{Re}\left[e^{-i\;\phi }\;b\right]$$

After inserting this $\hat{r}$ into (22), the estimator $\hat{\phi }$, which minimizes $\chi^{2}\left(\omega ,\;R_{2}^{*},\;\phi \right)$ for given ω and $R_{2}^{*}$, can also be calculated analytically [16]

(24) $$\hat{\phi }\left(\omega ,\;R_{2}^{*}\right)=\frac{1}{2}\;\mathrm{Arg}\left[b^{T}\;\text{Re}{\left[B\;\right]}^{-1}\;b\right]$$

such that the cost function

$$\chi^{2}\left(\omega ,R_{2}^{*}\right):=\chi^{2}\left(\omega ,\;R_{2}^{*},\;\hat{\phi }\left(\omega ,\;R_{2}^{*}\right)\right).$$

depends on ω and $R_{2}^{*}$ only, like in the unconstrained case.

There is one subtlety though, which makes this approach still potentially vulnerable: $r_{w},\;r_{f}$ are real, but not guaranteed to have the same sign

(25) $$r_{w}\cdot r_{f}\geq 0.$$

Indeed, violation of (25) implies the opposed phase of water and fat (7). In the following, we will therefore investigate an alternative approach, which allows to remove this vulnerability without having to sacrifice any of the benefits provided by VARPRO.

### 2.3 Fully Constrained: FC‐VARPRO

We start with a change of variables

(26) $$c_{w}=:c\;\left(1-f\;\right)\;c_{f}=:c\;f,$$

assuming f∈ℝ and 0≤f≤1. Solving for the new variables yields

(27) $$c=c_{w}+c_{f}\;f=\frac{\left|\;c_{f}\;\right|}{\left|\;c_{w}\;\right|+\left|\;c_{f}\;\right|}$$

The data model (10) then becomes

(28) $${ŝ}_{j}=c\;\left[1+f\;w_{j}\right]\;e_{j},$$

with

(29) $$w_{j}:=\beta_{j}-1,$$

and the minimization (14) is replaced by

(30) $$\hat{\omega },\;{\hat{R}}_{2}^{*},\hat{f},\;ĉ=\underset{f\;\in \;[0,1]}{\text{argmin}}\chi^{2}\left(\omega ,\;R_{2}^{*},\;f,\;c\right).$$

We now apply VARPRO with respect to the unconstrained complex coefficient c only, such that the new one‐column matrix

(31) $$A_{j1}:=\left[1+f\;w_{j}\right]\;e_{j},$$

depends on ω, $R_{2}^{*}$ *and* f.

This is not yet fully satisfying, since we would like to remove the dependence on f from the VARPRO cost function

(32) $$\chi^{2}\left(\omega ,\;R_{2}^{*},\;f\right)=s^{2}-b^{*}B^{-1}\;b,$$

as was achieved [16] for ϕ in the real‐weighted case before.

### 2.4 Removal of the Fat Fraction Dependence

To this end, we proceed in an analogous fashion:

For fixed ω and $R_{2}^{*}$, we search for $f\left(\omega ,R_{2}^{*}\right)$, which minimizes $\chi^{2}$ subject to the constraint f∈[0,1].

With (31) and (17), we first note that

(33) $$b^{*}B^{-1}\;b=\frac{z_{2}\;f^{2}+2\;z_{1}\;f+z_{0}}{n_{2}\;f^{2}+2\;n_{1}\;f+n_{0}},$$

with the abbreviations

(34) $$\begin{array}{ll} z_{2} & :={\left|\;u^{*}s\;\right|}^{2}\\ z_{1} & :=\text{Re}\left[\left(s^{*}u\right)\left(e^{*}s\right)\right]\\ z_{0} & :={\left|\;e^{*}s\;\right|}^{2}, \end{array}$$

(35) $$\begin{array}{ll} n_{2} & :=u^{*}u\\ n_{1} & :=\text{Re}\left[e^{*}u\right]\\ n_{0} & :=e^{*}e \end{array}$$

and the vector u given by $u_{j}:=w_{j}\;e_{j}$.

Taking the derivative with respect to f then leads to

(36) $$\frac{\partial \chi^{2}}{\partial f}=g\left(f\right)\cdot \left[a_{2}\;f^{2}+2\;a_{1}\;f+a_{0}\right]$$

where

(37) $$\begin{array}{ll} a_{2} & :=z_{1}\;n_{2}-z_{2}\;n_{1}\\ a_{1} & :=\frac{1}{2}\left[z_{0}\;n_{2}-z_{2}\;n_{0}\right]\\ a_{0} & :=z_{0}\;n_{1}-z_{1}\;n_{0} \end{array}$$

and g(f) is strictly positive everywhere. The condition

(38) $$\partial \chi^{2}/\partial f=0,$$

can only be achieved if the bracket on the RHS of (36) vanishes. If there is such a solution $f_{*}$, which satisfies all of the following conditions

- $f_{*}$ is real
- $0\leq f_{*}\leq 1$
- $\partial^{2}\chi^{2}/\partial f^{2}\left(f_{*}\right)>0$ (local minimum)

then

(39) $$f\left(\omega ,R_{2}^{*}\right)=f_{*}$$

In all other cases, there is no local minimum inside the interval [0,1] and we must either choose f=0 or f=1:

(40) $$f\left(\omega ,R_{2}^{*}\right)=\underset{f\;\in \;\{0,1\}}{\text{argmin}}\chi^{2}\left(\omega ,\;R_{2}^{*},\;f\right).$$

Inserting this $f\left(\omega ,\;R_{2}^{*}\right)$ on the RHS of (32) finally leads to the desired cost function

(41) $$\chi^{2}\left(\omega ,R_{2}^{*}\right):=\chi^{2}\left(\omega ,R_{2}^{*},f\left(\omega ,R_{2}^{*}\right)\right),$$

which does not depend on f anymore.

### 2.5 Multiple Coil Elements

Before comparing the three VARPRO variants, let us, similar to Stinson et al. [8], also briefly consider the case when independent signals of $n_{c}$ different coil elements (indexed by γ) are available, such that the multicoil version of (28) becomes

(42) $${ŝ}_{j\gamma }=c_{\gamma }\;\left[1+f\;w_{j}\right]\;e_{j}.$$

The linear coefficients $c_{\gamma }$ now also include information about the relative coil sensitivities and phases. In addition, with the data given by $s_{j\gamma }={ŝ}_{j\gamma }+\eta_{j\gamma }$, we now also have to look at the noise term $\eta_{j\gamma }$ more carefully than in the combined case above. The coil noise covariance matrix Ψ (which can be measured independently, for example during sequence calibration) is defined by

(43) $$\begin{array}{ll} \left\langle \;\eta_{j\gamma }\;\eta_{j^{\prime }\gamma^{\prime }}^{*}\;\right\rangle & =:\delta_{jj^{\prime }}\cdot \Psi_{\gamma \gamma^{\prime }}\\ & =:\underset{k}{\sum }\;\sigma_{k}^{2}\cdot U_{\gamma k}\;U_{\gamma^{\prime }k}^{*}. \end{array}$$

In the second line, we expressed the Hermitian, positive definite matrix Ψ by an eigendecomposition, such that the kth column of the unitary $n_{c}\times n_{c}$ matrix U (notation: $U_{\gamma k}^{*}\equiv {\left[U^{*}\right]}_{k\gamma }$) is the eigenvector to eigenvalue $\sigma_{k}^{2}>0$.

The multicoil ML cost function

$$\chi^{2}=\underset{\gamma ,\gamma^{\prime },j}{\sum }\;\left[s_{j\gamma }^{*}-A_{j1}^{*}\;c_{\gamma }^{*}\right]\;{\left[\Psi^{-1}\right]}_{\gamma \gamma^{\prime }}\;\left[s_{j\gamma^{\prime }}-A_{j1}\;c_{\gamma^{\prime }}\right],$$

can therefore be diagonalized

(44) $$\chi^{2}=\underset{k}{\sum }\;{\left\|\;s_{k}-A\;c_{k}\;\right\|}_{2}^{2},$$

by the transformation of data

(45) $${\left[s_{k}\right]}_{j}:=\underset{\gamma }{\sum }\;\frac{1}{\sigma_{k}}\;U_{\gamma k}^{*}\;s_{j\gamma },$$

and linear coefficients

(46) $${\left[c_{k}\right]}_{1}:=\underset{\gamma }{\sum }\;\frac{1}{\sigma_{k}}\;U_{\gamma k}^{*}\;c_{\gamma }.$$

Note that A is still defined by (31).

The whole analysis of the previous subsection, therefore, remains valid if we replace (34) by

(47) $$\begin{array}{ll} z_{2} & :=\underset{k}{\sum }\;{\left|u^{*}s_{k}\right|}^{2}\\ z_{1} & :=\underset{k}{\sum }\;\text{Re}\left[\left(s_{k}^{*}\;u\right)\left(e^{*}s_{k}\right)\right]\\ z_{0} & :=\underset{k}{\sum }\;{\left|e^{*}s_{k}\right|}^{2}. \end{array}$$

Again, we use the resulting fully‐constrained VARPRO cost function (41) to first determine estimators of ω and $R_{2}^{*}$, from which the linear coefficients are obtained subsequently via (20) and (17)

(48) $${ĉ}_{k}={\left(A^{*}A\right)}^{-1}A^{*}\;s_{k}.$$

With these ${ĉ}_{k}$, we can then undo the transformation (46) to estimate the coil‐dependent coefficients ${ĉ}_{\gamma }$.

## 3 Methods

### 3.1 Implementation

An implementation of the three VARPRO models is freely available in the Julia package B0Map [17].

### 3.2 Equidistant Echo Times

Although the above formalism is not limited to this case, we will, in the following, focus on equidistant (ΔTE) echo times and set φ:=ω·ΔTE. This allows to discuss the 2π‐periodic cost function $\chi^{2}\left(\varphi ,R_{2}^{*}\right)$ instead of $\chi^{2}\left(\omega ,R_{2}^{*}\right)$.

### 3.3 In‐Vivo Datasets

The different VARPRO variants were tested on GRE datasets, acquired with four, three, and two echo times:

#### 3.3.1 Four Echoes

A 2D dataset [18] (coronal head/neck region) from the 2012 ISMRM Fat Water Challenge, acquired at 1.5T with minimal echo time ${\text{TE}}_{\min}=1.35\;\text{ms}$ and echo spacing ΔTE=2.3ms.

#### 3.3.2 Three Echoes

A coronal 3D head/neck dataset from a previous graph‐cut study [9], acquired at 3T, with three monopolar echoes $\left({\text{TE}}_{\min}=1.06\;\text{ms},\Delta \text{TE}=1.59\;\text{ms}\right)$. A large FOV ($480\times 480\times 224\;{\text{mm}}^{3}$) was covered with resolution 2×2×4mm.

#### 3.3.3 Two Echoes

An abdominal 3D dataset (FOV: $350\times 350\times 252.8\;{\text{mm}}^{3}$ with 2.5mm isotropic resolution, TE=1.0,2.1ms, TR=3.4ms) from a previously proposed relaxometry technique [19].

The three‐ and two‐echo measurements were performed at 3T (Ingenia Elition X, Philips Healthcare, The Netherlands) using a 16‐channel anterior coil and 12‐channel posterior coil. All volunteer scans were approved by the local institutional review board (Klinikum rechts der Isar, Technical University of Munich, Munich, Germany) and informed consent was obtained from the volunteer as part of the previous studies.

### 3.4 Data Fitting

For each VARPRO model, the global minimum of $\chi^{2}\left(\varphi ,R_{2}^{*}\right)$ was determined similar to [20]:

1. Assuming equidistant echo times, the search interval φ∈(−π,π] was split into four nonoverlapping intervals $I_{j}$ of equal size. For each interval, a golden section search (GSS) was conducted to determine the minimum
(49) $$\varphi_{j}=\underset{\varphi \;\in \;I_{j}}{\text{argmin}}\;\chi^{2}\left(\varphi ,\;R_{2}^{*}=0\right).$$
2. For each such determined $\varphi_{j}$, the associated relaxation rate $R_{2j}^{*}$ was determined with another GSS search
(50) $$R_{2j}^{*}=\underset{R_{2}^{*}}{\text{argmin}}\;\chi^{2}\left(\varphi_{j},\;R_{2}^{*}\right).$$
3. Using them as starting points, each such determined pair $\left(\varphi_{j},R_{2j}^{*}\right)$ was further refined by nonlinear minimization of $\chi^{2}$.
4. The final phase and relaxation rate estimates are given by the combination $\left(\varphi_{j},R_{2j}^{*}\right)$ which gives the minimal $\chi^{2}$.

Steps 2 and 3 were skipped for the two‐echo dataset, for which we made the usual assumption [8, 16] $R_{2}^{*}\equiv 0$ to avoid underdetermined fitting.

## 4 Results

**FIGURE 1:**
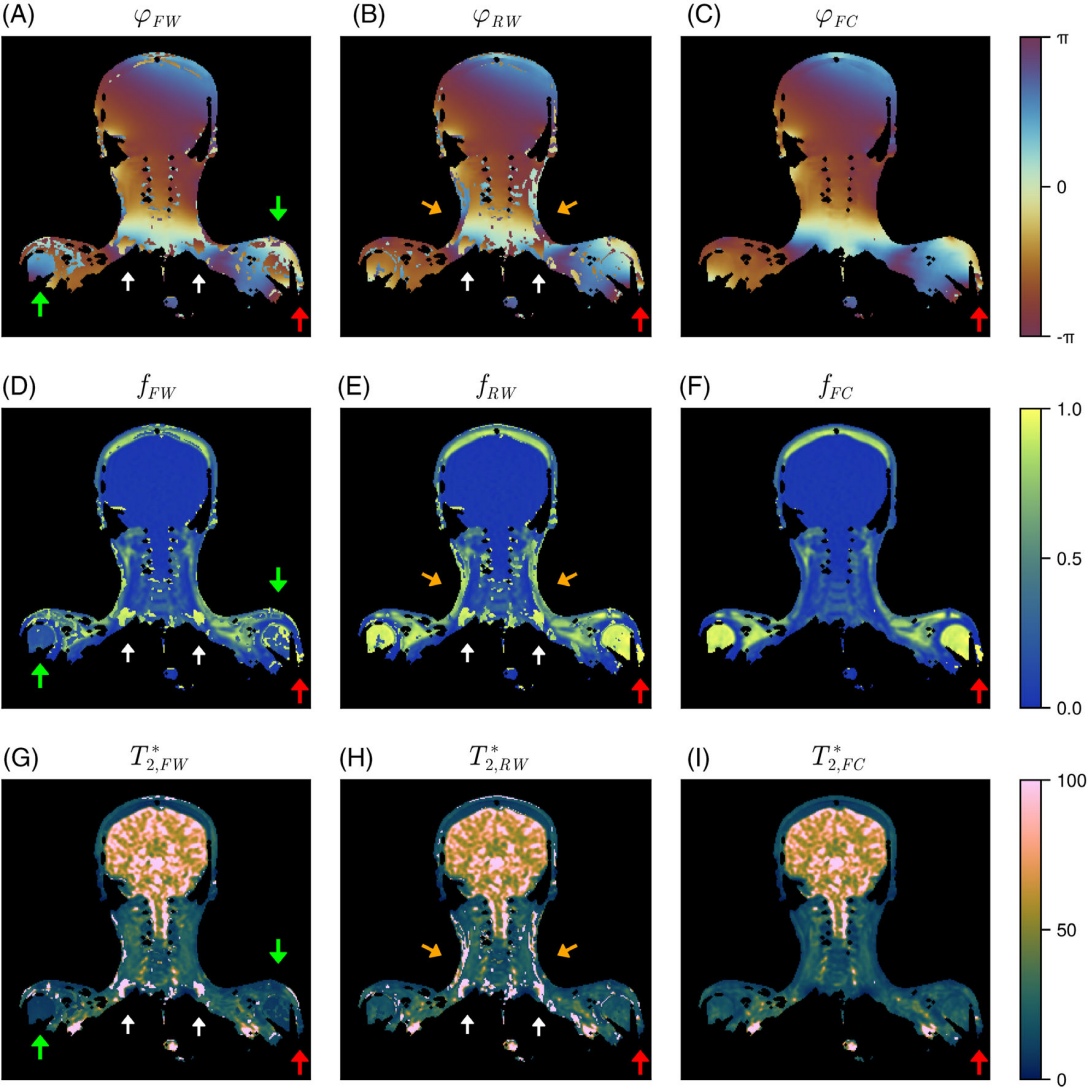
Four echo scan (${\text{TE}}_{\min}=1.35\;\text{ms}$, ΔTE=2.3ms) at 1.5T from the 2012 ISMRM Fat Water Challenge. Shown is the fitted phase (first row), PDFF (second row), and $T_{2}^{*}$ (third row). Compared to unconstrained VARPRO (left column), real‐weighted VARPRO (middle column) mainly resolves problems in the Humerus region (green arrows). Other regions, particularly above the lung apex (white arrows), remain problematic and partly new errors emerge (orange arrows). Except for a remaining problem (red arrow) at the edge of the FOV, fully‐constrained VARPRO (right column) resolves these issues.

**FIGURE 2:**
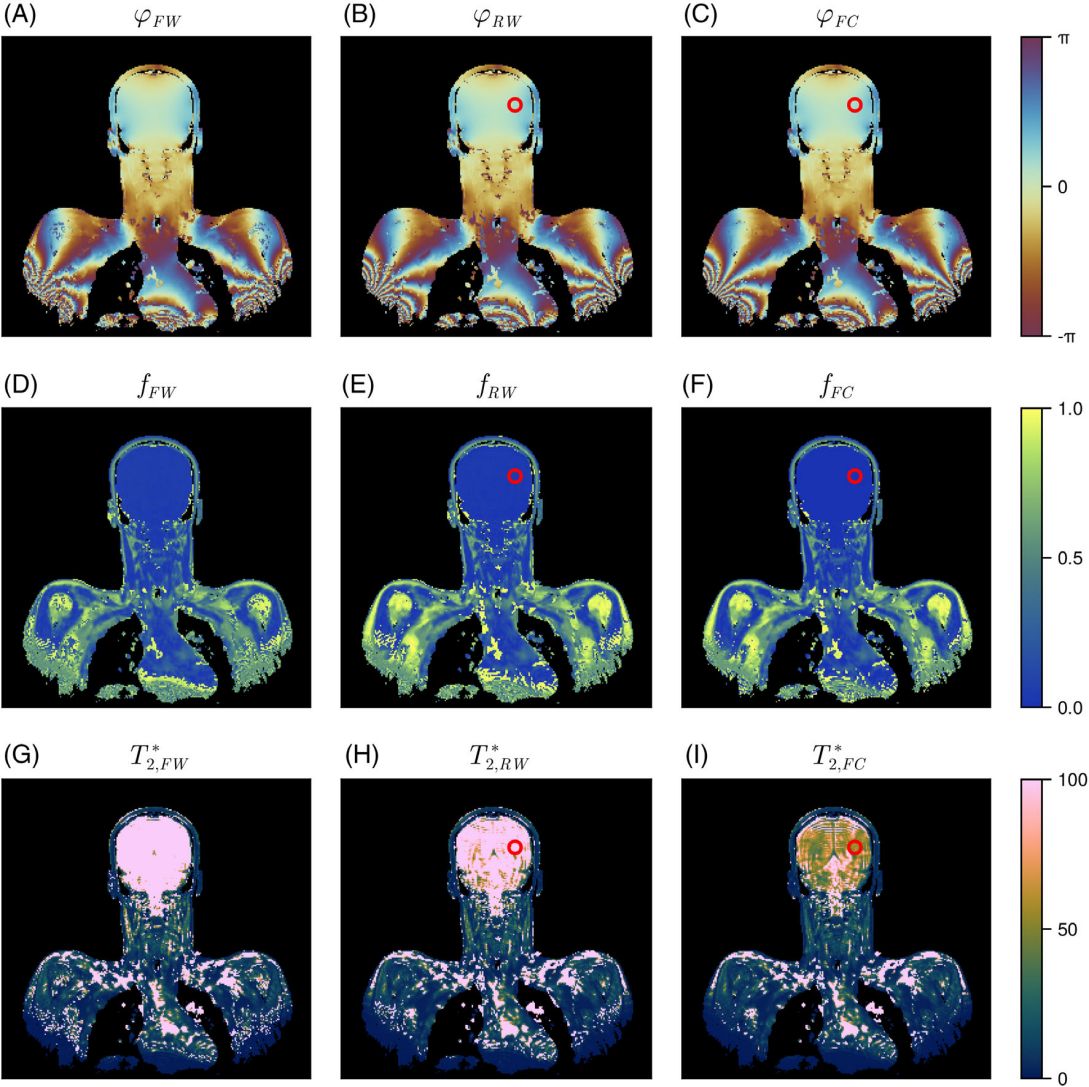
Same maps as in Figure 1, now for a three echo (${\text{TE}}_{\min}=1.06\;\text{ms}$, ΔTE=1.59ms) GRE scan, acquired at 3T. Differences between RW‐ and FC‐VARPRO are most apparent in the $T_{2}^{*}$ maps of the brain. For example, the pixel in the center of the red circle is fitted by $\left(\varphi ,T_{2}^{*},f\right)\approx \left(27.6^{\circ },120.8\;\text{ms},0.034\right)$ (RW) and $\left(26.9^{\circ },52.6\;\text{ms},0\right)$ (FC), respectively. Also, compared to the four echo scan in Figure 1, the overall reliability of purely local fitting as such is generally reduced—particularly below the neck, where $B_{0}$ is rather inhomogeneous.

Figure 1 (four echoes), Figure 2 (three echoes) show the best local fit of phase φ, fat fraction f, and $T_{2}^{*}$ for the three different VARPRO variants.

**FIGURE 3:**
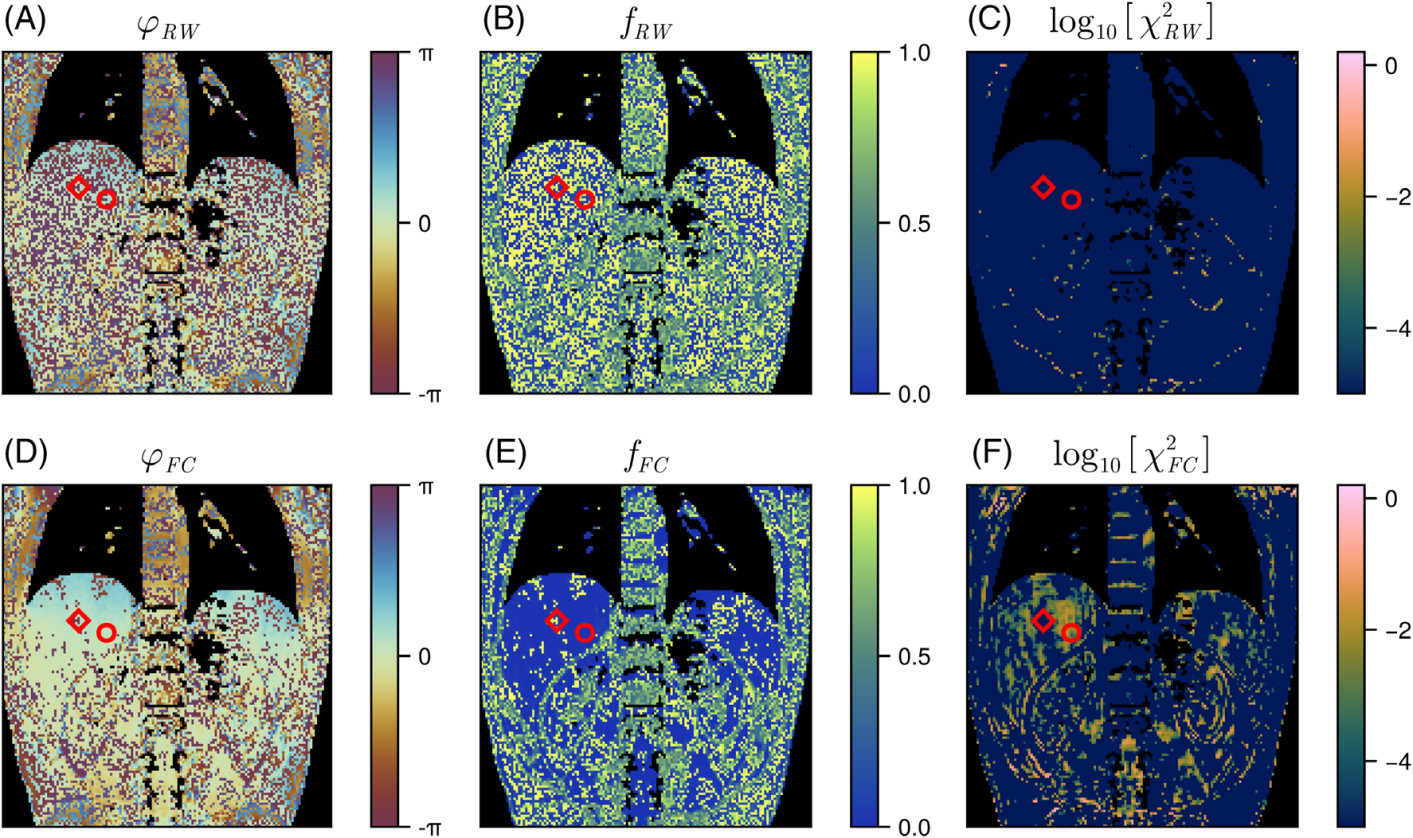
Coronal two‐echo (TE=1.0,2.1ms) thoracal‐abdominal GRE at $B_{0}=3\;T$. As the $\chi^{2}$ map **C** shows, RW‐VARPRO (first row) fits the data perfectly almost everywhere. Since these solutions are not unique, the associated parameter maps (**A**, **B**) are numerically unstable though. When the in‐phase condition (25) holds (e.g., pixel in the center of the red diamond), RW‐VARPRO and FC‐VARPRO become equivalent, and the same problems consequently arise in the fully‐constrained model calculations (**D**, **E**) as well. In the opposed‐phase situation (7), FC‐VARPRO predicts a pure tissue ($f_{FC}=0$ for the pixel in the center of the red circle in **E**). In such cases, the corresponding fit is no longer perfect ($\chi_{FC}^{2}>\chi_{RW}^{2}$ in **F**), but the parameter maps **D** and **E** become more stable.

For two‐echo GRE (Figure 3), FW‐VARPRO is omitted, because unconstrained weights $c_{w},c_{f}\in \mathbb{C}$ lead to overfitting: $\chi_{FW}^{2}\left(\varphi ,R_{2}^{*}\right)\equiv 0$. Furthermore, no $T_{2}^{*}$ is displayed, since the two constrained VARPRO versions assume $R_{2}^{*}\equiv 0$.

**FIGURE 4:**
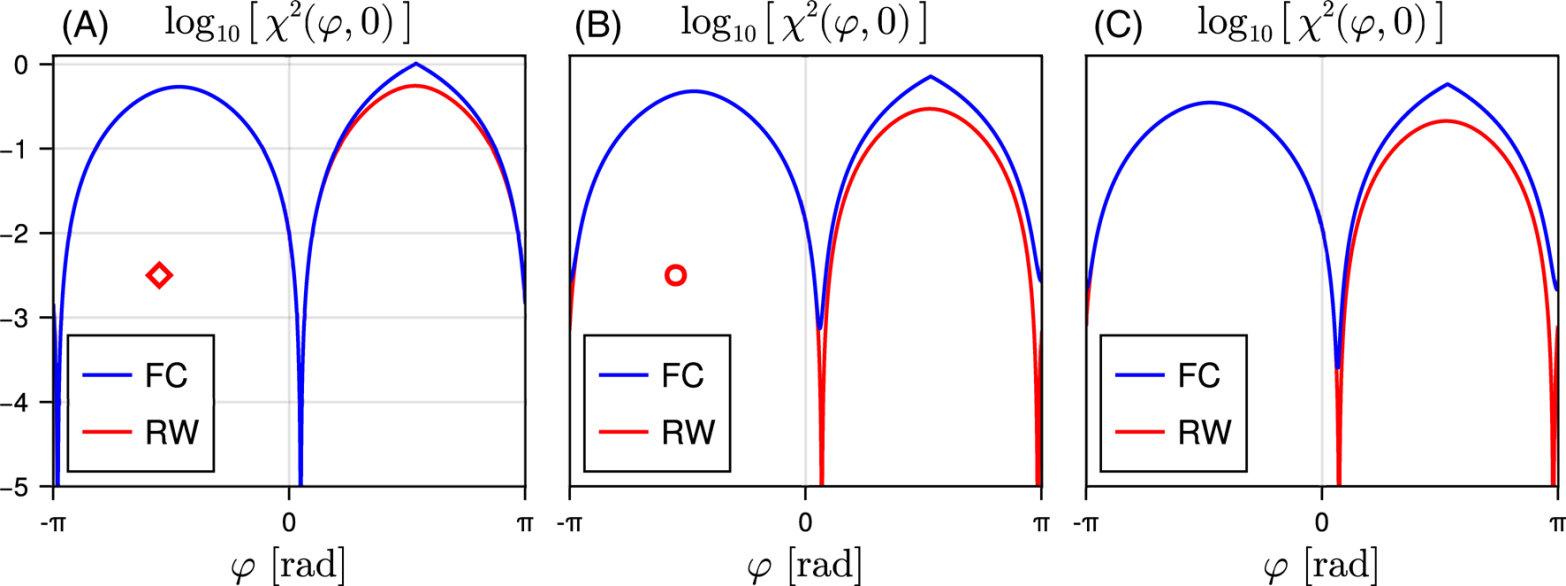
For the two‐echo scan of Figure 3, exemplary cost functions $\chi^{2}\left(\varphi ,\;R_{2}^{*}=0\right)$ are displayed (logarithmically, to visually emphasize perfect fitting) with RW‐VARPRO in red and FC‐VARPRO in blue. Subplot **A** was calculated with the in‐phase data from the center of the red diamond in Figure 3. There are two equivalent solutions, which perfectly fit the data in both VARPRO variants and which solution is actually chosen in Figure 3 is essentially the result of a numerical coin toss. Subplot **B** corresponds to the opposed‐phase case (red circle in Figure 3). Here, the minima of RW‐VARPRO and FC‐VARPRO differ: Still, the former (red) produces two perfect fits, whereas the latter (blue) does not, and there is a unique global minimum. This case can be nicely reproduced in a noise‐free simulation (**C**), which was based upon f=0.02 and $T_{2}^{*}=20\;\text{ms}$ to resemble liver tissue. (For better comparability, phase and amplitude were adapted to subplot **B**.)

In all scans, the proposed FC‐VARPRO clearly performs best, followed by RW‐VARPRO, which mostly (exception: orange arrows in Figure 1) improves upon FW‐VARPRO. The two constrained VARPRO versions only differ, where RW‐VARPRO violates (3):

- In Figure 1 (four echoes), this leads to phase offsets, due to ending up in the wrong local minimum of $\chi_{RW}^{2}$. Corresponding to confusion of water and fat peak, this also affects the PDFF and $T_{2}^{*}$.
- In Figure 2 (three echoes), the correct minimum is usually found. Here, opposed‐phase solutions (7) rather lead to an overestimation of $T_{2}^{*}$, particularly in the brain.
- Figure 3 shows that local fitting with only two echoes is inherently unstable. This is due to the fact that usually two distinct solutions perfectly fit the data if two species (water and fat) are available. (cf. Appendix A for a brief explanation)
  In such cases, stable results can only be obtained where RW‐VARPRO violates (25), since then FC‐VARPRO is restricted to fitting with a pure tissue (water or fat). A confirming numerical analysis is provided in Figure 4.

**FIGURE 5:**
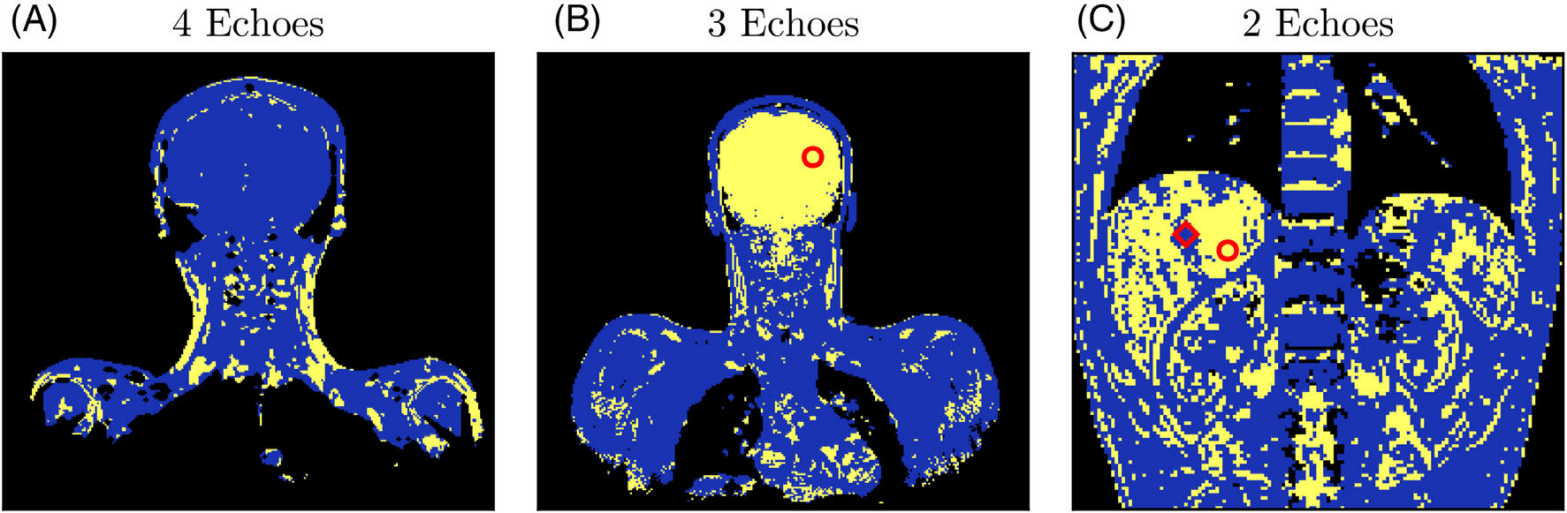
The pixels where RW‐VARPRO violates the in‐phase condition (25) in Figures 1, 2, 3 are marked in yellow.

Figure 5 shows where RW‐VARPRO differs from FC‐VARPRO in the three in‐vivo examples.

## 5 Discussion

Combining local data consistency $\chi_{\rho }^{2}$ with regularization ℛ in a total cost function (1) is the most common approach to analyze multi‐echo GRE data in the context of water‐fat separation. The need to adhere to physically motivated phase constraints (3) requires some attention though:

1. In the conventional maximum likelihood (ML) formulation (2), this can be accomplished by constraining the search space of the linear parameters $c_{w\rho }$ and $c_{f\rho }$.
2. Due to the elimination of the latter, this is not possible for VARPRO, and the validity of (3) therefore depends on how linear parameters are defined in the underlying data model:
  FW‐VARPRO ((10), (11), (12)): Treating $c_{w\rho }$ and $c_{f\rho }$ as free complex coefficients (usually) violates (3).
  RW‐VARPRO (21): With two real‐valued coefficients $r_{w\rho }$ and $r_{f\rho }$, either (3) (in‐phase) or (7) (opposed‐phase) is possible.
  FC‐VARPRO ((26), (27), (28)): With a single complex prefactor $c_{\rho }$, the in‐phase constraint (3) is always fulfilled.

The considered in‐vivo scans indeed confirmed the importance of obeying the correct initial condition.

Where RW‐VARPRO satisfies the in‐phase condition (3) and (25), it is equivalent to FC‐VARPRO: $\chi_{RW}^{2}\left(\varphi ,R_{2}^{*}\right)\equiv \chi_{FC}^{2}\left(\varphi ,R_{2}^{*}\right)$.

Where RW‐VARPRO violates (25), FC‐VARPRO cannot find a local minimum (39) for any actual water‐fat mixture 0<f<1. While FC‐VARPRO deals with this situation by selecting the best pure tissue (40), RW‐VARPRO enters the forbidden range and searches for an opposed‐phase local minimum (7).

This can lead to two types of adverse consequences:

a. Since $\chi_{RW}^{2}<\chi_{FC}^{2}$ in the opposed‐phase regime, minimization of $\chi_{RW}^{2}$ is more likely to end up in an incorrect local minimum than minimization of $\chi_{FC}^{2}$. Corresponding to a confusion of water and fat, this type of error is typically associated with a visible phase jump and incorrect PDFF values. (Also $T_{2}^{*}$ is often affected.)
b. Where RW‐VARPRO violates (25), corresponding local minima of $\chi_{RW}^{2}$ and $\chi_{FC}^{2}$ are slightly displaced in the $\left(\varphi ,R_{2}^{*}\right)$ plane. If the echo train duration is short compared to $T_{2}^{*}$, this can have a significant impact on the resulting $T_{2}^{*}$ maps, even if the same (in the sense of corresponding) local minimum is found. In the in‐vivo scans, these effects were particularly observable in the brain (Figure 2).

Errors of type (a) are usually resolved by regularization. Here, since the initial guess is less affected by confusion of water and fat, FC‐VARPRO can help to improve and stabilize the convergence of optimization algorithms—particularly, in case of poor data quality (low SNR, inaccurate tissue model).

Contrary to that, regularization should not be effective for errors of type (b), since the correct local minimum of $\chi^{2}$ has already been found. Here, the in‐phase condition (3) turned out to be particularly important for accurate $T_{2}^{*}$ maps. In the context of VARPRO, this can only be guaranteed by the fully‐constrained version.

Two‐echo GRE is a special case, since it is underdetermined, even for high SNR:

Actual (0<f<1) mixtures of water and fat often generate two perfect fits, where $\chi^{2}$ vanishes exactly. One solution corresponds to the correct identification of water and fat peaks, while the other one relates to error type (a) above.

Sometimes, such instabilities in RW‐VARPRO can be resolved in FC‐VARPRO:

When RW‐VARPRO violates (25), only a pure tissue can be used for fitting the data in FC‐VARPRO and $\chi^{2}$ becomes more meaningful in terms of identifying the correct local minimum. The areas where FC‐VARPRO outperforms RW‐VARPRO can therefore be expected to improve stability and convergence of regularized approaches (1).

## 6 Conclusion

Fully‐constrained VARPRO guarantees parallel alignment of water and fat components at TE=0 and thereby removes instabilities, which were occasionally observed in previously published VARPRO variants.

The derived closed‐form expressions allow for a numerically efficient implementation of local data consistency $\chi_{\rho }^{2}$, which is compatible with any regularized cost function (1).

## Funding

The authors have nothing to report.

## Disclosure

The authors have nothing to report.

## Conflicts of Interest

The authors declare no conflicts of interest.

## Data Availability

The code used to produce the figures in this article is available: Ganter [21]. The 3D datasets can be obtained from the corresponding author upon reasonable request.

## Appendix A Two‐Echo Solutions

VARPRO with two echoes is special, since it often allows for exact solutions.

In case of free complex weights, (17) simply reduces to $\hat{c}=A^{-1}\;s$, such that (15) vanishes identically for any ω and $R_{2}^{*}$, as long as the (quadratic) matrix A has full rank.

With real weights, we obtain $\chi_{RW}^{2}=0$, iff the solution (23) can be written in the form

(A1)
$$\hat{r}=e^{-\;i\;\phi }\;A^{-1}\;s$$

For this, it is apparently necessary (and sufficient) that the imaginary part on the RHS vanishes:

(A2)
$$\text{Im}\left[e^{-\;i\;\phi }\;A^{-1}\left(\varphi ,R_{2}^{*}\right)\;s\right]=0$$

For fixed $R_{2}^{*}$ (otherwise, (A2) is underdetermined), this condition can be rewritten as a set of two (j=1,2) equations

(A3)
$$\begin{array}{ll} \left[a_{j}\left(s\right)\;\cos\frac{\varphi }{2}+b_{j}\left(s\right)\;\sin\frac{\varphi }{2}\right]\cdot \cos\Psi & +\\ \left[c_{j}\left(s\right)\;\cos\frac{\varphi }{2}+d_{j}\left(s\right)\;\sin\frac{\varphi }{2}\right]\cdot \sin\Psi & =0, \end{array}$$

with Ψ:=ϕ+αφ (the real constant α only depends on the echo times) and real‐valued functions $a_{j}$, $b_{j}$, $c_{j}$ and $d_{j}$. The two equations can be used to eliminate Ψ and, after further regrouping, we finally end up with a condition of the form

(A4)
x(s)·cosφ+y(s)·sinφ+z(s)=0,

again with real‐valued functions x, y and z.

Since (A4) is 2π‐periodic, it has two solutions on (−π,π]—if solutions exist at all. (The degenerate case with one solution, when (A4) coincides with an extremum of the LHS, has zero measure.)

Perfect fitting of data, if present, therefore typically occurs for two distinct parameter sets (φ,ϕ) in RW‐VARPRO. If the data s do not allow for a solution of (A4), the minimum of $\chi_{RW}^{2}$ becomes nonzero (cf. bright areas in Figure 3C).

The same applies to FC‐VARPRO, if RW‐VARPRO satisfies the in‐phase condition (25) (since the two variants are equivalent then). Only when RW‐VARPRO violates (25), a unique minimum can be found in FC‐VARPRO.
